# Aluminum Doping Effects on Interface Depletion Width of Low Temperature Processed ZnO Electron Transport Layer-Based Perovskite Solar Cells

**DOI:** 10.3389/fchem.2021.795291

**Published:** 2022-01-05

**Authors:** Muhammad Adnan, Muhammad Usman, Saqib Ali, Sofia Javed, Mohammad Islam, Muhammad Aftab Akram

**Affiliations:** ^1^ Department of Materials Engineering, School of Chemical and Materials Engineering, National University of Sciences and Technology, Islamabad, Pakistan; ^2^ Department of Materials Science and Engineering, College of Engineering, Peking University, Beijing, China; ^3^ New Mexico Institute of Mining and Technology, Socorro, NM, United States

**Keywords:** ZnO thin films, low temperature, Mott Schottky analysis, perovskite solar cells, charge transport, charge recombination, electron transparent conducting oxide

## Abstract

Rapid improvement in efficiency and stabilities of perovskite solar cells (PSCs) is an indication of its prime role for future energy demands. Various research has been carried out to improve efficiency including reducing the exciton recombination and enhancement of electron mobilities within cells by using electron transport material (ETM). In the present research, electrical, optical, and depletion width reduction properties of low temperature processed ZnO electron transport layer-based perovskite solar cells are studied. The ZnO thin films vary with the concentration of Al doping, and improvement of optical transmission percentage up to 80% for doped samples is confirmed by optical analysis. Reduction in electrical resistance for 1% Al concentration and maximum conductivity 11,697.41 (1/Ω-cm) among the prepared samples and carrier concentration 1.06×10^22^ cm^−3^ were corroborated by Hall effect measurements. Systematic impedance spectroscopy of perovskite devices with synthesized ETM is presented in the study, while the depletion width reduction is observed by Mott Schottky curves. IV measurements of the device and the interfacial charge transfer between the absorber layer of methylammonium lead iodide and ETM have also been elaborated on interface electronic characteristics.

## Introduction

To cope with recent energy demands, the role of solar cells is attaining much attention (Iqbal et al., 2012; Tsarev et al., 2021; Ying et al., 2021). The need of the hour is to shift towards sustainable and green energy products (Midilli et al., 2006). Due to the increased human population and growth in energy consumption, the world’s energy resources are depleting day by day. The dependence on fossil fuels like natural oil and coal gas is exceeding by an alarming rate (Höök and Tang, 2013; Russo, 2021; Zipper et al., 2021). Also, the burning of these materials produces gases like CO_2_, SO_x_, and NO_x_ which are not only causing pollution in the environment but also threatening the human race’s survival by causing global warming and greenhouse effects (Raval and Ramanathan, 1989). Green energy production is the cardinal matter in the context of natural resources preservation which opens up the avenues for synthesis of ubiquitous potential materials for energy harvesting applications (Balaprakash et al., 2018). Nanotechnology is arguably the technology of century (Roco, 2011): Few notable properties that might change or behave differently for nanomaterials than bulk materials are optical (Djurisic and Leung, 2006), electrical, magnetic (Slonczewski, 1961; Adnan et al., 2021; Rabbani et al., 2021), mechanical strength, charge storage capacity, biological activities (Usman et al., 2020), fluorescence, magnetic permeability, chemical reactivity (Tao, 2008), and melting points.

Many materials are being exploited which do not only provide renewable energy but are also cost-effective (Dincer, 2000; Raza et al., 2020; Usman et al., 2021). The need of the hour is to prepare environment-friendly and green products so that these energy harvesting devices do not pollute the environment. Since then, many materials have been synthesized, and many devices have been fabricated like solar cells (Yamaguchi et al., 2005), wind energy, fuel cells, capacitors, and supercapacitors which not only can produce renewable energy but also can store energy for a long power run. The increased usage of energy and reliance on portable energy devices is demanding increased energy and power density.

Perovskites were first effectively used in solid-state solar cells in 2012 (Yamaguchi et al., 2005); these types of solar cells contain perovskite structured material such as hybrid organic and inorganic material as an absorber layer for photovoltaics properties (Ramanathan et al., 2003; Qiao et al., 2019; Liu et al., 2020). Perovskite material is used as a photon harvesting material, whilst for charge transportation, different materials are used. At the interface of charge transport material and perovskite, the separation of exciton occurs. For electron transport, material only permits electrons and blocks the holes and vice versa for hole transport materials (Lin et al., 2018); these layers of material are also termed as ETL and HTL, respectively. The perovskite solar cells are a contender for the lead role of energy conversion devices of future generations. Their promising energy conversion feature with the low-cost manufacturing made them the most persuaded topic of research and exploring the new materials for electron transport layer/materials is advancing over time.

These materials are specifically used as electron extractors from the perovskite absorber materials. The basic principle which is being applied is bandgap alignment, i.e., the conduction and valance band in the case of inorganic semiconductors, while HOMO and LUMO of organic materials are as such that electron travels across the high potential, while Holes are being blocked. Historically, the rapid increment in the efficiency for the perovskite-based solar cells somewhat depends on the higher transfer of electron transport materials which imitates the purpose of exploring the new, efficient, and cost-effective electron transport materials; the key factors which might be the priority to use some materials for the electron transporting layer are higher electron mobility, high surface area, compatible bandgap; a sufficient energy band gap is preferable which restricts the electron-hole recombination.

These are compounds have metallic and oxide ions, and metal oxides are basic in chemical natures (Mann and Watson, 1958). Metal oxides have been widely studied for their properties spanning from electrical conductivity (Shahid et al., 2016) and catalytic activity (Iqbal et al., 2012) to biological activities (Riaz et al., 2019); their abundance is one of the most significant factors to consider the metal oxides for applications.

Many metal oxides such as ZnO (Gupta, 1990), TiO_2_ (Mughal et al., 2019), SnO_2_, and ZnSO_4_ have been utilized as an electron transport layer for better extraction of charge (Akimoto, 2012). Also, various research groups have studied core-shell nanoparticles TiO_2_/MgO (Karuppuchamy and Brundha, 2015), Al_2_O_3_/ZnO, and WO_3_/TiO_2_ for charge blockage, and enhanced charge recombination resistance by such material which is an insulator with effective charge deployment and charge transports. The state-of-the-art PSCs employed mesoporous metal oxide scaffolds, mostly TiO_2_, which requires a high-temperature annealing process (≈500 °C) which limits their use for flexible substrates, while the next widely studies ETL is SnO_2_ which has oxidation and morphological issues. So, to reduce the mentioned issues, the ZnO-based ETLs are studied in this literature.

Zinc oxide has 60 meV exciton binding energy (Aftab Akram et al., 2016) and fast electron mobility. ZnO is a semiconductor material with a direct bandgap (3.11–3.36 eV), while its higher exciton binding energy makes it ideal for several purposes in different fields such as its nanoparticle-based films for solar cell, light-emitting diodes, spintronics devices, and sensors for gas detection. The lattice parameters of ZnO are as follows; unit cell area = 3.24 Å and lattice constant = 5.1 Å. Their ratio (c/a ∼1.60) is optimum value for hexagonal unit cell c/a = 1.633. Its bonding is ionic in nature (Zn^2+^–O^2−^) with ionic radii of 0.75 Å for Zn^2+^ and 1.30 Å for O^2−^. Crystal structures of zinc oxide are rock salt (Razavi-Khosroshahi et al., 2017), zinc blende, and wurtzite. The preferable crystal structure for better electrical properties is wurtzite.

It is also stated that the effect on electrical resistivity by surface modification of ZnO and doped ZnO-based ETL is observed, which also incorporated the bandgap tuning, enhancement of optical transmission, and the effect of surface morphology on other factors such as light-harvesting of absorber material and electron transport.

## Experimentation

For the substrate soda-lime glass and FTO coated glass was used. Initially, the fluorine doped tin oxide coated glass slides were rinsed with soap water several times, then in bath sonicator treated with acetone, ethanol, and deionized water for 15 min respectively and dried after each washing. To attain better charge conductivity and for compact films adherence, the FTOs were annealed at 150°C for 15 min (Salam et al., 2013).

The sol-gel method was brought into consideration for the synthesis of ZnO. Solution (0.2 mol) of zinc acetate hexahydrate (Zn(CH_3_COO).6H_2_O, Sigma-Aldrich >99.0%) into iso-propanol was reported in literature (Salam et al., 2011). The continuous stirring was done for homogenous mixing. After the complete dissolution of precursor, monoethonalamine was added dropwise for stabilizing of reaction. It acts as a capping agent to ZnO nanoparticles in solution. After this, solution was kept for ageing for 24 h at room temperature. Afterward, the aged solution was used for thin-film formation.

For aluminum doping into zinc oxide, two solutions were prepared of 0.2 M aluminum chloride di-hydrate in ethanol and 0.2 M zinc acetate hexahydrate in isopropanol. For doping appropriate volume of aluminum solution into zinc solution, stirring was done for the complete mixture. Afterward, samples were labeled as zinc oxide is i-ZnO, 1 at% Al-doped ZnO as 1% Al, 2 at% Al-doped ZnO as 2% Al, and 3 at% Al-doped ZnO as 3% Al and annealed at 150 °C for 1 h.

## Characterization

The morphology and mapping images of synthesized material were obtained using a scanning electron microscope (SEM JSM6490, JEOL). The XRD patterns of samples were recorded using an X-ray diffractometer (Bruker D8 ADVANCE) with Cu Kα (*λ* = 1.5406 Å) radiation. The lattice constants of as-prepared films were calculated by X’Pert HighScore software. Ultraviolet-visible (UV-vis) absorption spectra of various ZnO/AZO films were characterized on Jenway UV-73 Series spectrophotometer in the wavelength range of 350–800 nm. The J-V curves were recorded by a solar simulator developed by SCEINCETECH Co., Ltd. under AM 1.5G simulated solar illumination (100 mW/cm). The electrochemical characterization was carried on Potentiostate Biologic VSP.

## Results and Discussion

**FIGURE 1:**
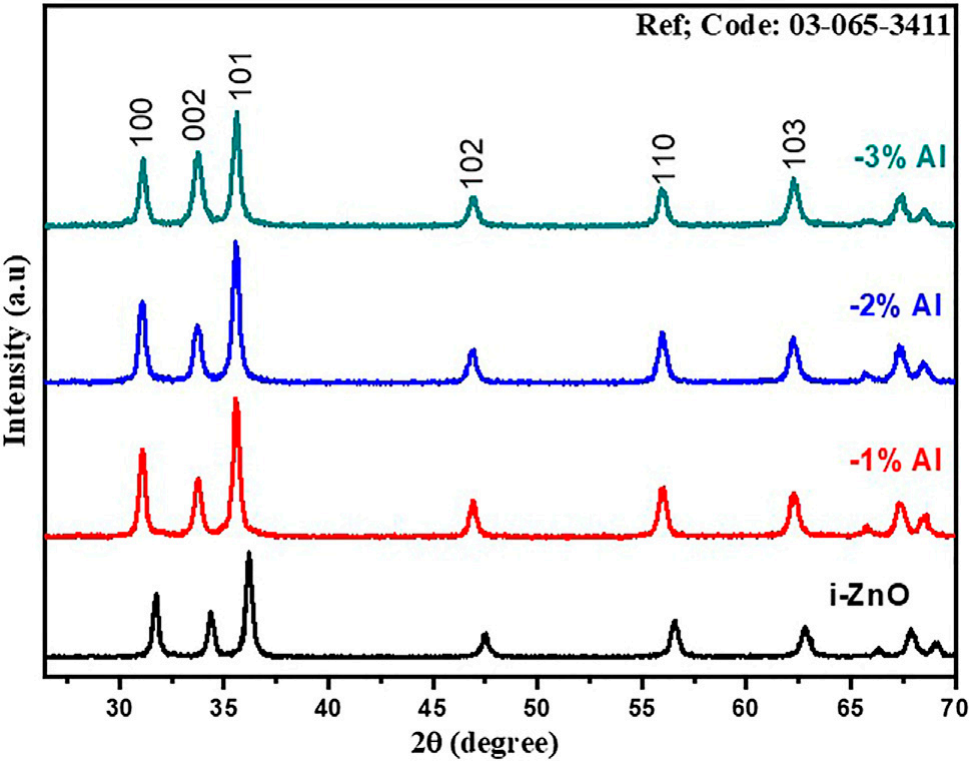
XRD spectra of ZnO and Al doped ZnO nanomaterials.

The identification of crystal structure and study of crystallographic planes were carried out by X-ray diffraction (XRD). Figure 1 represents the XRD patterns for undoped ZnO and Al-doped ZnO nanomaterials. The interpretation and analysis of patterns were carried out with scrupulous attention and gingerly as it is the foremost technique for confirmation of doping. From the analysis of XRD data, the hexagonal wurtzite crystal structure having space group P63mc of ZnO was confirmed and matched with JCPDS 01-089-1397 (Suwanboon et al., 2008). In addition to this, XRD patterns of Al-doped ZnO at various concentrations affirm that no change in the wurtzite crystal structure occurs as there were no additional peaks. Upon details analysis, by addition of Al in ZnO, a left volumetric shift among peaks was observed. In XRD patterns for Al doping, peaks are shifting toward left i.e., θ is decreasing which suggests an increase in d spacing between planes. For further detailed structural studies of ZnO lattice, constant values were estimated; by using unit cell software, a was 3.2529Å and c was 5.21299Å, while entire cell volume was found 47.7726 Å for 1% Al-doped sample. As ionic radii of Al^3+^ is 0.53Å which is fewer than ionic radii of Zn^2+^ which is 0.74Å so substitutional doping of Al^3+^ in ZnO produces shrinkage of the lattice which is because of left volumetric change was found in XRD patterns. For spin-coated films, this gives crystallite size value in the range of 16–20 nm. The crystallite size for intrinsic zinc oxide film is found 18 nm approximately, for 1% Al-doped slightly changes to 20 nm, while for 2% Al-doped found 22 nm and 3% Al-doped it is estimated in 18–20 nm range and shown in Table 1. In Figure 1, the XRD patterns for intrinsic ZnO are shown. These XRD patterns confirm the hexagonal crystal structure having 100 planes at 2 θ= 31.02, 002 planes at 33.75, 101 planes at 35.6075, 102 planes at 46.98, 110 planes at 55.98, while 103 planes at 62.105.

**TABLE 1:** Average Crystallite size of samples.

Sample name	Crystallite size (nm)
Intrinsic ZnO	18
1% Al doped ZnO	20
2% Al doped ZnO	22
3% Al doped ZnO	20

Characterization of atomic vibrational study of Al-doped ZnO is done by FTIR. In Supplementary Figure S1, it has been observed that for intrinsic ZnO, 1% Al-doped and 2% Al-doped weak absorption spectra at wavenumber 398 cm^−1^, 406 cm^−1^, and 414 cm^−1^ are detected which are referring to Zn-O-Zn. Also, there are stretching at 1383 cm^−1^ because of the stretching vibration of Zn-O-Zn interfered by Al atoms. Moreover, asymmetric vibration of the Zn-O-Zn bond varies because of uptake of Al-O-Al (Balaprakash et al., 2018). For ZnO FTIR spectra, spectra are matched by reference, and it was found that weak absorption spectra at wavenumber 398cm^−1^, 406cm^−1^, and 414 cm^−1^ are detected which are referring to Zn-O-Zn. The FTIR spectra of 1% Al-doped is showing peaks 398 cm^−1^, 406 cm^−1^, and 414 cm^−1^ which are because of Zn-O-Zn absorption spectra, while the extra peak at 1383 cm^−1^ which is because of stretching vibration of Zn-O-Zn interfered by Al atoms which are residing in lattice (Bai et al., 2013).

Similarly, the FTIR spectra of 2% Al-doped is showing peaks 398 cm^−1^, 406 cm^−1^, and 414 cm^−1^ which are because of Zn-O-Zn absorption spectra, while the extra peak at 1383 cm^−1^ is because of stretching vibration of Zn-O-Zn interfered by Al atoms which are residing in the lattice.

Also, further doping has no significant influence on the spectra; for 3% Al-doped ZnO FTIR spectra are showing peaks 238 cm^−1^, 298 cm^−1^, and 414 cm^−1^ which are because of Zn-O-Zn absorption spectra, while the extra peak at 1383 cm^−1^ and 1621 cm^−1^ is because of stretching vibration of Zn-O-Zn interfered by Al atoms which are residing in the lattice.

**FIGURE 2:**
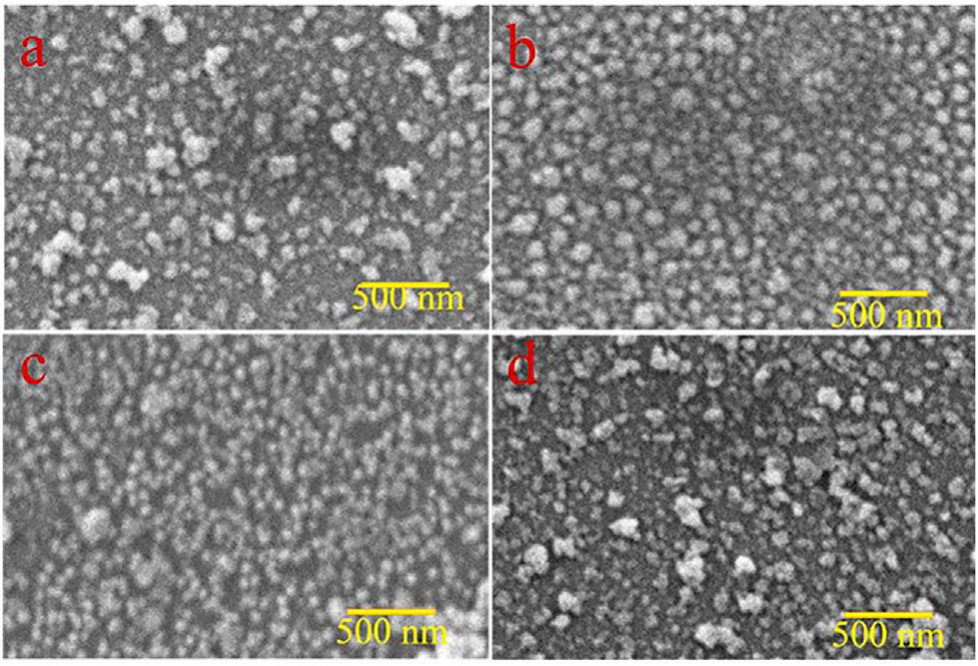
Top view SEM images **(A)** ZnO thin film, **(B)** 1% Al doped ZnO, **(C)** 2% Al doped ZnO d) 3% Al doped ZnO.

For the analysis of surface modification, scanning electron microscopy (SEM) is used. The top view SEM images of ZnO, 1%Al, 2% Al, and 3% Al thin films are shown in Figure 2. The SEM analysis suggests the film uniformity and smoothness are gradually improved for 1 and 2% of Al doping concentration in ZnO. These properties render to the synthesis technique and parameters of film thickness; further, films are spin-coated and parameters like spin speed and sample loading are kept almost constant. The images show a relatively fully covered area. The EDS spectra of doped samples are shown in Supplementary Figure S5.

**FIGURE 3:**
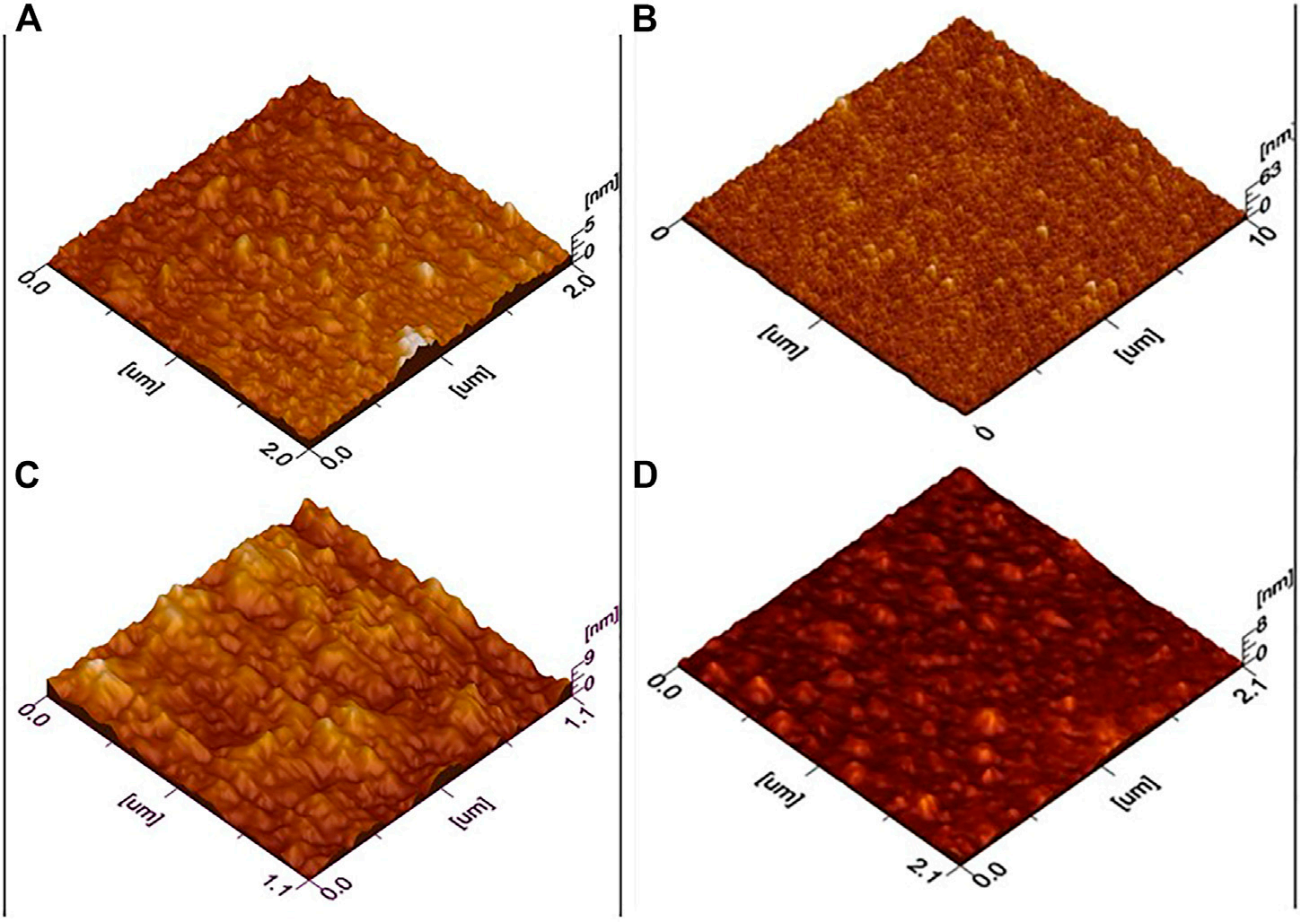
Three-dimensional AFM images of **(A)** ZnO, **(B)** 1% Al, **(C)** 2% Al, and **(D)** 3% Al.

For morphological analysis and roughness measurements, AFM images of the sample were obtained, and 3D AFM images are shown in Figure 3. For ZnO, it can be interpreted by the image that nanoparticles are homogenously distributed in films. Furthermore, film thickness studies show uniformity across all scanned areas. Root mean square roughness was estimated 10.4 nm. For 1% Al-doped ZnO film smoothness is evident from the results. Also, in some areas, there is a slight agglomeration of particles which might be because of fewer nucleation sites available and time of reaction as reported in the literature (Salam et al., 2013). The root means square roughness found to be 7.2 nm which decreasing with doping. Similarly, for 2% Al & 3% Al, no such differences are observed from the first two samples except for roughness increasing trend and for 3% Al particles are more agglomerated and film uniformity is slightly disturbed. The surface roughness for pure ZnO is 1.53 nm which is corroborated by XRD results as crystallite size was calculated by XRD patterns is 18 nm which results in uniform particle size and nucleation at the interface is rapid which also helps in the uniformity of particle’s distribution as low surface roughness. For 1%, 2%, and 3% Al-doped ZnO was found 2.43, 2.34, and 2.12 nm.

All these results were in comparison of XRD results as crystallite size for 1% Al-doped ZnO was calculated by XRD patterns is 20 nm which is slightly higher than intrinsic ZnO which results in less uniform particle size and although nucleation at the interface is rapid which also helps in uniformity of particle’s distribution but due to Al doping lattice shrinkage results in larger grains which causes surface defects and higher surface roughness. As the doping percentage increase, the surface morphology is changing, though the doping percentage is very less an increase in crystallite size results in broader particle size, this causes a slight variation in surface roughness, and it increases. Using AFM in contact mode, surface roughness was found 2.78 nm as crystallite size was calculated by XRD patterns is 28 nm which is slightly higher than intrinsic ZnO which results in less uniform particle size and although nucleation at the interface is rapid which also helps in uniformity of particle’s distribution but due to Al doping lattice shrinkage results in larger grains which causes surface defects and higher surface roughness. Also, the other factors which involve the sol viscosity and sample treatment have played an important role in the film roughness, as sample preparation was carried out by spin coating which involves the spin speed, acceleration, and deceleration speed of spin coater. These factors have a significant effect on the surface morphology as well as the temperature of annealing has played a pivotal role in homogenous film formation. These roughness properties were corroborated by the electrical properties such as charger carrier concentration and resistivity.

**FIGURE 4:**
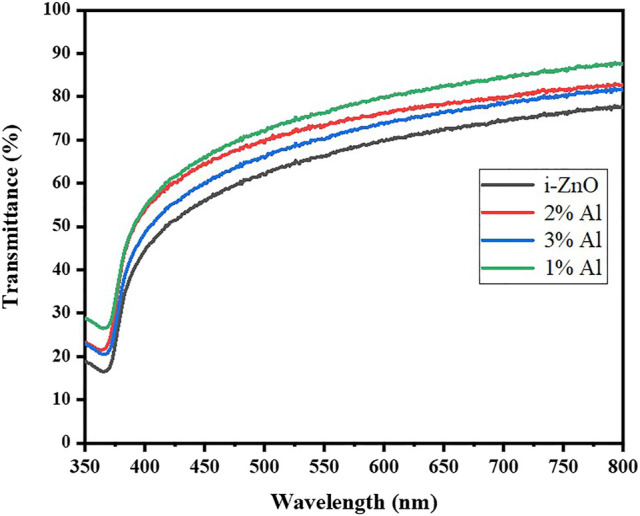
Optical transmittance spectra of ZnO and Al doped ZnO thin films.

Ultraviolet-visible (UV Vis) spectroscopic studies were carried at room temperature; the relation of transmittance in correspondence to wavelength is shown in Figure 4. It shows a change of transmittance at the Al doping. As the doping concentration increases, the transmittance follows the blue shift trend monotonically. However, upon further doping decrease of transmittance is evident and it follows the trends for further doping of aluminum (Djurisic and Leung, 2006). This is one of the traits of Al substitutional doping as it resides in Zn defects and reduces oxygen vacancy in the lattice which was initially causing the green and yellow emission (Mani Rahulan et al., 2019). For band gap measurements from Uv Vis Spectra tauc plot method was used and a bandgap for intrinsic Zinc oxide was found 3.11 eV; for 1% Al-doped zinc oxide it was slightly higher 3.20 eV, 2% Al 3.21 eV and for 3% Al it was 3.24 eV as Shown in Table 2. The change in bandgap arise as donor impurity was introduced in the lattice which donated electrons in the conduction band resulting shift of Fermi level towards conduction band. In the present case, the Fermi level shift towards conduction band termed as blue shift causes the increase in the bandgap of Al-doped ZnO which increases with uprising concentration of dopant.

**TABLE 2:** Energy Band gap of samples.

Sample name	Band gap (eV)
i-ZnO	3.11
1% Al	3.20
2% Al	3.21
3% Al	3.24

**FIGURE 5:**
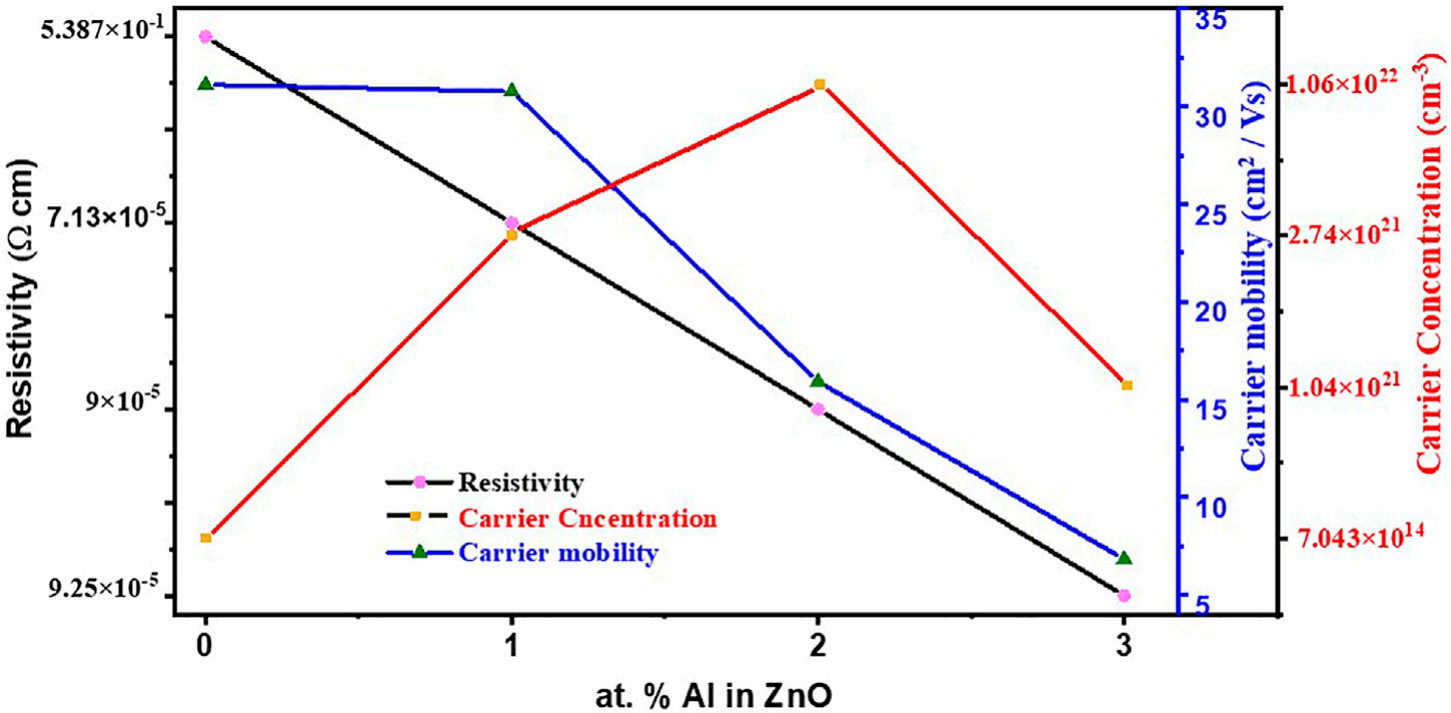
Variation in resistivity, carrier mobility, and carrier concentration of ZnO films with Al doping.

Electrical properties were measured by the DC Hall effect apparatus and shown in Figure 5. Swin system of 5300G magnetic field was employed in the system, testing was carried out at a 300 K temperature and dark. Hall effect measurements of prepared samples were carried out to study the electrical properties of materials; specifically, the establishment of electric current through prepared films, the effect of doping concentration on the three main parameters which defines the characteristics of electrical behaviors was evident and by far had a discreet difference. In Supplementary Table S1, sheet resistance (ohm/sq), resistivity (ohm-cm), conductivity (1/ohm-cm), carrier concentration (cm^−3^), and sheet carrier mobility (cm^2^/Vs) of ZnO undoped and Al-doped thin films are illustrated. Resistivity is directly proportional to sheet resistance by the following relation

Rs=ρ/T (1)

Here, R_s_ is sheet resistance,  ρ is resistivity, and T is the thickness of the film. Thickness was measured by EM which was found in the nanometer range. Similarly, conductivity is reciprocal of resistivity.

α=1/ρ (2)

Here α is conductivity which varies inversely with resistivity. It is noted that electrical properties depend on carrier concentration and sheet resistance (Manifacier et al., 1979). Both properties are interlinked with Al doping, in which Al^3+^ resides on substitutional sites of Zn^2+^, Zn, and Al interstitial atoms and oxygen vacancies. For Al doping, the change in resistivity is drastic.

For intrinsic ZnO, it has a value of 5.387 × 10^−1^ (ohm-cm) which decreases to an appreciable value of 7.13 × 10^−5^(ohm-cm) for 1% Al doping. This is because Al^3+^ replaces Zn^2+^ in lattice sites which ultimately increases carrier concentration.

Upon further Al doping in ZnO, slight increase in resistivity is observed with increasing doping concentration. One the reason which might be suggested for this phenomenon is that Al has limited solubility in ZnO and, upon further doping, Al^3+^ might form Al_2_O_3_ and ZnAl_2_O_4_ which are non-conductive in nature and accumulate at grain boundaries causing high resistivity.

In present work that electrical properties such as resistivity and carrier concentration for Al-doped ZnO thin films were reported, the properties were improved from previously reported values because of pretreatment, improvement in film surface defects control, and several time bettered results are obtained by pre-treating substrate at 150°C for initiation of nucleation at sites and better film growth. Similarly, annealing of samples was carried out in an inert atmosphere which helped in the reduction of oxygen vacancies and fewer impurities.

The sheet resistance of the sample denoted by Rs is determined by measuring characteristic resistance R_A_ and R_B_ with the four-probe method by the placement of the sample in the sample holder and connecting the four edges (Chwang et al., 1974). Van der Pauw equation for the relation of parameters is given below (Look and Molnar, 1997)

$$\exp\left(-\rho \;R_{A}/R_{s}\right)+\exp\left(-\rho \;R_{B}/R_{R}\right)=1$$ (3)

Also, charge carrier densities and charge mobilities are determined by this technique by applying a series of voltage measurements across the test specimen, while the current and magnetic field constant are kept constant.

Samples were prepared in a 1 × 1 cm^2^ area and tested in the same size in the Hall effect measurement apparatus. Sheet resistance for intrinsic ZnO is 5.986 × 10^4^ Ω/sq which is reduced to 8.07 Ω/sq for 1% Al doping, as by doping the Al^3+^helps in the supply of more charge carriers.

Further, for 2% Al sheet resistance is increased slightly to 8.57 Ω/sq and with 3% al doping sheet resistance now starting to increase up to 8.91 Ω/sq which might be because of Al atoms which are excessive and doping limit is complete so they tend to make aluminates which are insulator in nature and causing of increasing the sheet resistance (Salam et al., 2011).

## Device Fabrication and Characterization

**FIGURE 6:**
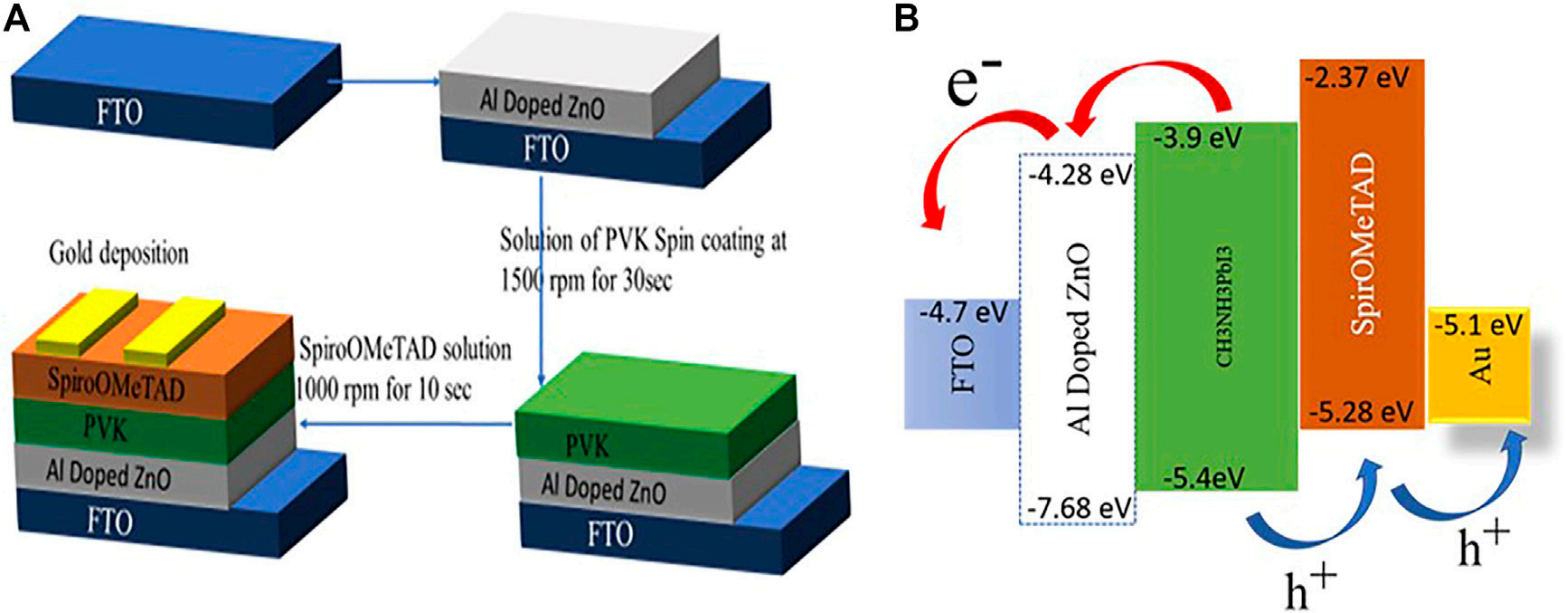
**(A)** Systematic representation of fabrication of device. **(B)** Energy band diagram of device.

For solar cell fabrication, FTO/glass was etched with zinc powder and HCL to obtain the required patterns and properly rinsed with detergent, DI water, acetone, and isopropyl alcohol respectively with a duration of 10 min each and. The solar cell structure was planar in which afterwards the washing patterned FTO/glass substrates were coated with ETL followed by absorber material and HTL as systematic is shown in Figure 6. ZnO,1% Al, and 2% Al films were coated on patterned FTO *via* spin coating. These films primarily act as ETL. On the ETL layer, CH_3_NH_3_PbI_3_ *via* two-step method acts as an absorber layer for solar cell: synthesized *via* hot injection method was spin-coated and Spiro-OMeTAD was used as HTL.

**FIGURE 7:**
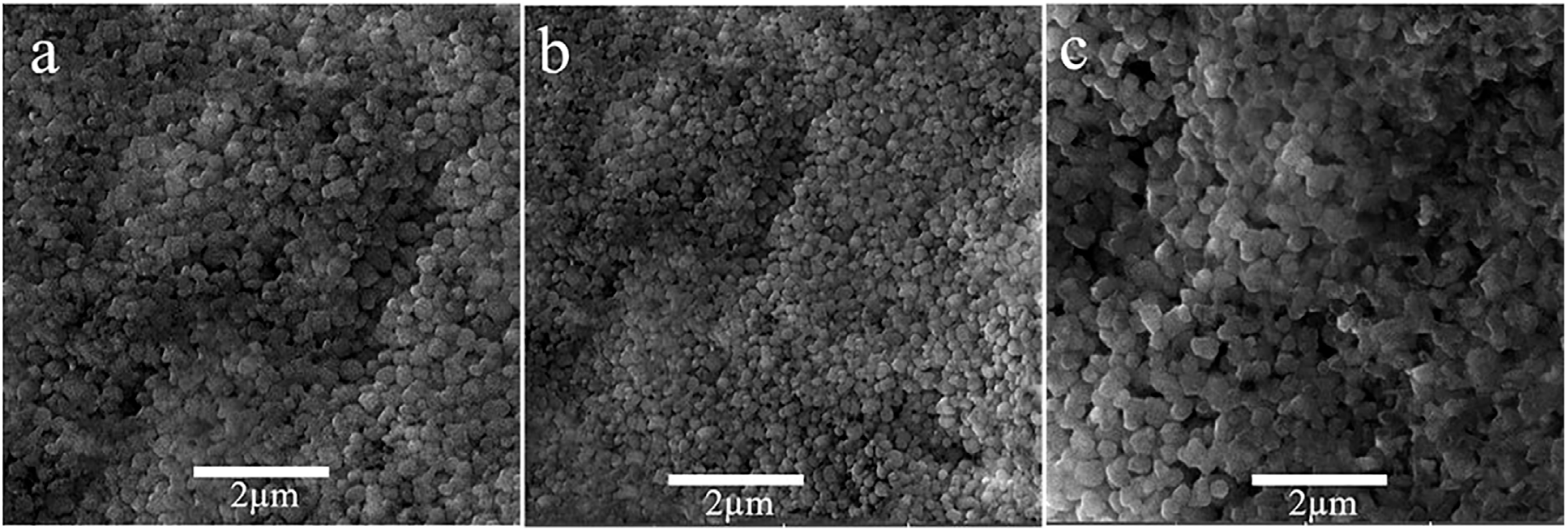
SEM top view of perovskite layer deposited on **(A)** ZnO ETL, **(B)** 1% Al, and **(C)** 2% Al.

The morphological analysis of the fabricated device is carried out by SEM. In Figure 7 SEM images are shown. These figures illustrate when the perovskite layer is deposited on ZnO. It has smaller grains and slight pin holes attributed to the nature of ZnO. Similarly with the addition of dopant slight change in grain size and smooth film morphology is observed. The cross-section image of the champion device is shown in Supplementary Figure S2.

**FIGURE 8:**
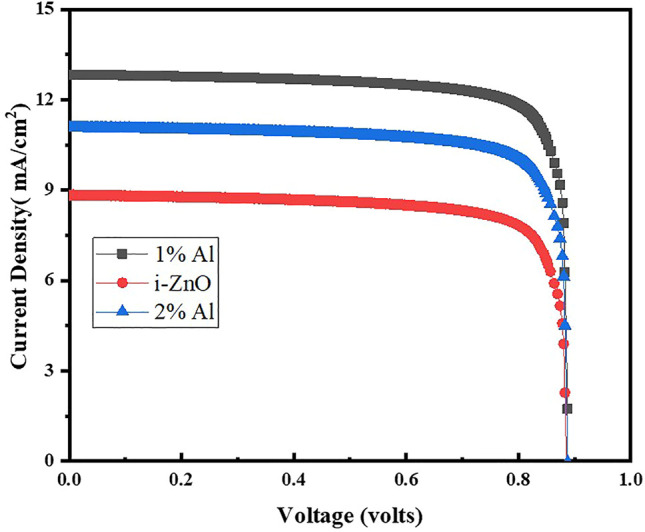
IV characteristics of fabricated cells.

The solar cell testing is carried out by using Science Tech. Solar cell testing mode is used for IV curves measurements, under light using the lamp having power100 mW. The cell area was 1 × 1 cm^2^. Figure 8 shows the device performance of the fabricated solar cells which are control samples of ZnO based ETL and Al-doped ZnO as ETLS. It shows the champion cell contains the 1% Al as ETL, having the PCE of 9.60% with J_sc_, V_oc_, and FF of 12.80 mA/cm^2^, 0.887 V, and 84.36%, respectively, which has been greater than previously reported single layer by Mahmood et al. (2014) and almost equal to double layer which is 10% efficient. Also, as reported by Zhang et al. (2015) electrodeposited ETL has produced efficiencies about 11%. As Liu and Kelly (2014) have reported and developed ZnO nanoparticle electron-transport layers for CH3NH3PbI3-based solar cells, they have demonstrated that neither effect of a mesoporous scaffold nor any high-temperature processing steps has a significant impact to achieve PCEs as high as 15.7%. This is expected to simplify dramatically the device fabrication procedures for such devices, but at the same time maintain or improve their already high device efficiency the present study involving the effect on interface depletion width of perovskite and electron transport layer and reduction in width as corroborated by the MS data with aluminum doping plays a vital role to understand the mechanism of exciton carrier’s movement within the cell.

For attaining a depth understanding of device performance in terms of recombination, interfacial charge transfer phenomena, and carrier transport within the device, we have performed electrochemical studies such as electrochemical impedance spectroscopy (EIS) and Mott-Schottky characterization of the complete device. It has been ensured that the electrical response is attained shortly after the fabrication of the device and not of the degraded device. Also, the device has retained the photovoltaics performance identical before and after the electrochemical analysis which asserts that these analyses are not influenced by the degradation of the device.

**FIGURE 9:**
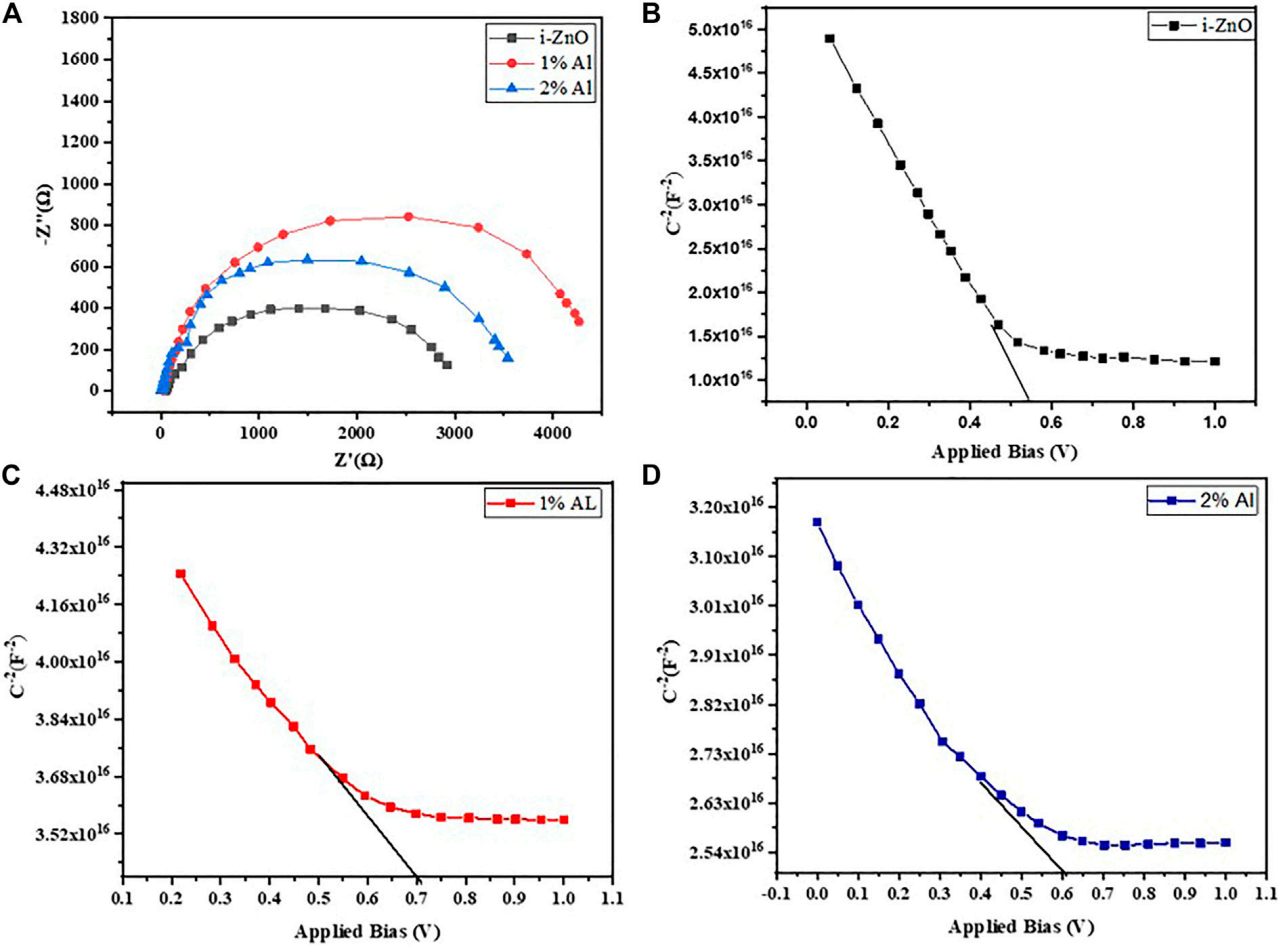
**(A)** Nyquist plot of perovskite devices and Mott Schottky curve at 10 kHz frequency for perovskite device with **(B)** ZnO ETL, **(C)** 1% Al ETL, and **(D)** 2% Al ETL.

Figure 9 shows the Nyquist plot for perovskite devices with ZnO, 1% Als, and 2% Al ETL performed at 700 mV under dark. From the amplified view of the high-frequency portion of the curves, we observe a small radius arc in high frequency which is followed by a larger radius arc for each device. Our experimental data fit nicely with a previously reported model (Liu et al., 2014) that used ZnO nanostructures as ETL in a perovskite solar device. The equivalent circuit consists of a series combination of one parallel R-C element and another parallel R-CPE (constant phase element) element combined with a series resistance, R_S_. In Table 3, R_C_, C_C_, R_Rec_, CPE, and R_S_ denote contact resistance and capacitance at ETL/perovskite or HTL/perovskite interface, recombination resistance, constant phase element originating from heterogeneity, and resistance incorporating metal contact and wire respectively. In the present work, the Spiro-oMetad is used as HTL, and metal contact resistance for all devices is almost identical 10 Ωcm2. The value for other parameters obtained from EIS data is given in Table 3.

**TABLE 3:** Electrical properties of samples.

ETL	R_c_ (Ω.cm^2^)	R_Rec_ (Ω.cm^2^)	C_c_(nF)	CPE-Y (nMho)	CPE-N
ZnO	11.27	74.45	2.23	65.23	0.52
1% Al	8.89	94	4.58	52.40	0.76
2% Al	12.01	64.09	1.16	68.05	0.45

There is a profound impact of flat-band potential and depletion width on device ultimate output. We can relate the device performance to these key device parameters with the information retrieved from the Mott-Schottky plot under dark conditions. The *x*-axis intercept of the extrapolated linear section of the Mott-Schottky curve gives the flat-band potential of the device, while the charge carrier density or doping density can be found from the slope of the curve using the following Eq. 4

$$N=\frac{\left(V_{fb}-V\right)C^{2}}{qA^{2}\epsilon }$$ (4)

Here V, V_fb_, C, A, q, and ε are applied bias, flat-band potential junction capacitance appearing due to the modulation in depletion width, active device surface area, elementary charge, and permittivity for perovskite respectively. The p-doped behavior of perovskite is evident (Bisquert and Garcia-Belmonte, 2011; Guerrero et al., 2016) from the Mott-Schottky curve lines. Since the dipping two-step fabrication technique of perovskite does not involve any annealing, the observation of p-type behavior from Mott-Schottky analysis complies with the report from the earlier literature (Mahmud et al., 2017). The perovskite layer tends to form P-N junction with adjacent ZnO and AZO layers, and Mott Schottky’s behavior should be contributed from both ETL and perovskite (Guerrero et al., 2014). So, the flat potential can be expressed as V_fb_ = V_fb_ (perovskite)+V_fb_ (ZnO). Figures 9B,C,D show the flat band potential and charge carrier density of the device obtained from the Mott Schottky curve. The overall flat band potential for the 1% champion device is 0.67 V, and the overall charge carrier density for the device was found 3. 60 × 1016. The flat band potential for ZnO, and 2% Al devices was found 0.55 and 0.60 V, respectively as shown in Table 4. Hence, all results obtained from different characterization techniques are consistent and show no anomaly, and bear testimony to the proper optimization of the Al-doped ZnO layer as an electron transport layer to be applied in methylammonium lead iodide perovskite Solar cell.

**TABLE 4:** MS data of samples.

ETL	Charge carrier density (cm^−3^)	Flat-band potential (V)	Depletion width (nm)
ZnO	1.63 × 10^16^	0.55	273.04
1% Al	3.60 × 10^16^	0.67	204.63
2% Al	2.58 × 10^16^	0.60	228.74

## Conclusion

In conclusion, we have reported low temperature (<150°C) wet chemical processed ZnO and Al-doped ZnO thin films as an electron transport layer with methylammonium lead iodide perovskite solar cell. This synthesis protocol provides a relatively cheaper and faster method of fabrication of solar cells. We have found that 1% Al-doped ZnO provides the most suitable ETM, antireflective electron extracting layer suppressing the deep trap states resulting in the decrease in depletion region width of perovskite and AZO (Mahmud et al., 2017), rendering to relatively better performance of device up to 9.60%. In the present work, we had used impedance spectroscopy to study the useful information regarding carrier generation, recombination, charge transport, and extraction in a perovskite solar device with synthesized ETM. Also, low contact resistance found from the Nyquist plot and thinner depletion width owing to high flat-band potential and larger carrier density from the Mott-Schottky curve indicate conforming ETL/perovskite interface for most efficient charge extraction.

## Data Availability

The original contributions presented in the study are included in the article/Supplementary Material. Further inquiries can be directed to the corresponding author.
