# Supplementary material for: Aluminum Doping Effects on Interface Depletion Width of Low Temperature Processed ZnO Electron Transport Layer-Based Perovskite Solar Cells
Source: Front Chem. 2022 Jan 5;9:795291. doi: 10.3389/fchem.2021.795291 (PMC8766970; doi:10.3389/fchem.2021.795291)
Supplement: Supplementary file 1 [file DataSheet1.docx]

**SUPPORTING INFORMATION**

**Aluminum doping effects on interface depletion width of Low temperature processed ZnO Electron transport layer-Based Perovskite Solar cells**

Figure S1: FTIR patterns of ZnO and Al doped ZnO Thinfilms

Table S1

|  | iZnO | 1% Al | 2% Al | 3% Al |
| --- | --- | --- | --- | --- |
| Sheet resistance (ohm/sq) | 5.986×10^4^ | 8.07 | 8.57 | 8.91 |
| Resistivity (ohm-cm) | 5.387×10^-1^ | 7.13×10^-5^ | 9.25×10^-5^ | 9×10^-5^ |
| Conductivity (1/ohm-cm) | 1.8587×10^0^ | 13492.93 | 11697.41 | 11360.05 |
| Carrier concentration (cm^-3^) | 7.043×10^14^ | 2.74×10^21^ | 1.06×10^22^ | 1.04×10^22^ |
|  |  |  |  |  |


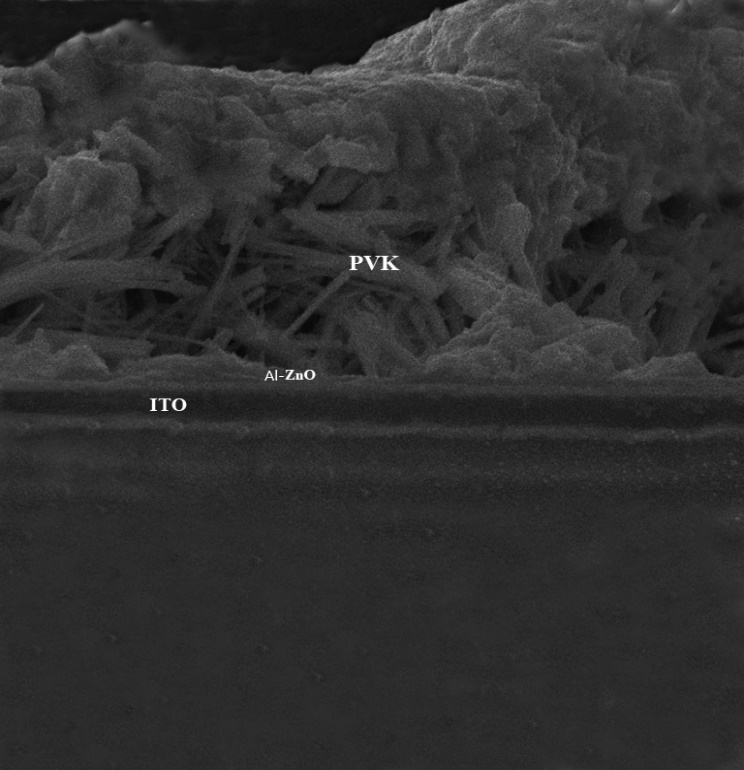


Figure S2: Cross Section of PVK layer


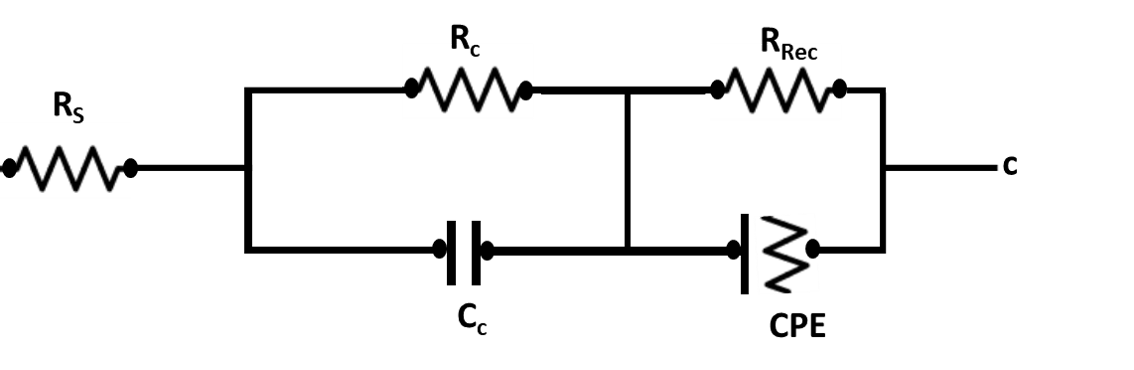


Figure S3: Equivalent circuit model used to fit the experimental data from Nyquist plot

Figure S4: Particle size distribution by DLS

*
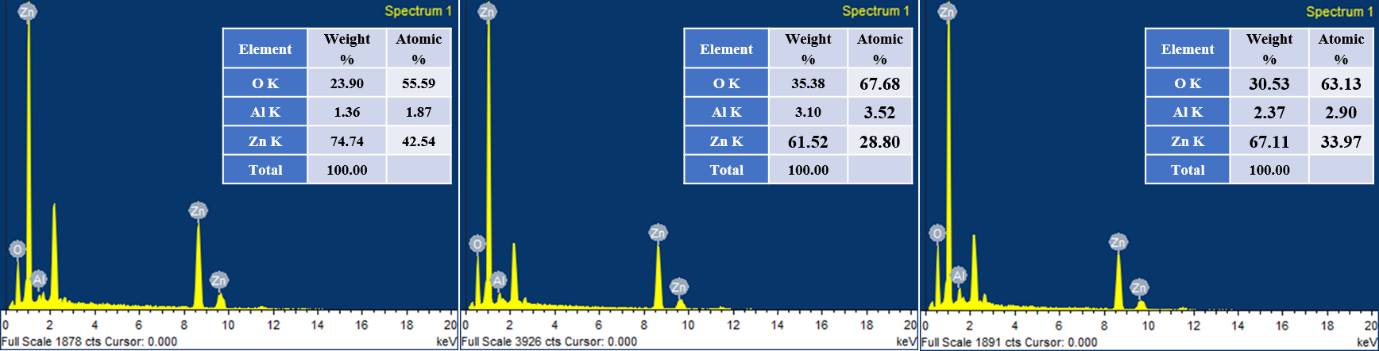
*Figure S5: EDS Spectra of 1% Al, 3% Al, 2% Al
